# Anesthesia, pain management and surgical approach of ovariectomy or orchiectomy in six Egyptian fruit bats (*Rousettus aegyptiacus*): A case report

**DOI:** 10.3389/fvets.2023.1121526

**Published:** 2023-03-13

**Authors:** Smadar Tal, Yael Shilo-Benjamini

**Affiliations:** ^1^Koret School of Veterinary Medicine, The Robert H. Smith Faculty of Agriculture, Food and Environment, The Hebrew University of Jerusalem, Rehovot, Israel; ^2^Department of Animal Sciences, Tel-Hai College, Qiryat Shemona, Israel

**Keywords:** Egyptian fruit bat, *Rousettus aegyptiacus*, anesthesia, analgesia, ovariectomy, orchiectomy

## Abstract

The purpose of this report is to describe the anesthetic and analgesic management and the surgical procedures of gonadectomy in six (four females and two males) healthy adult Egyptian fruit bats (*Rousettus aegyptiacus*). Bats were anesthetized with a combination of alfaxalone, midazolam, and morphine administered subcutaneously. Incisional line infiltration using bupivacaine was administered in all bats, and additional bilateral intratesticular injection was administered in the males. Ovariectomy was performed *via* a dorsal approach, by bilateral midline skin incisions at the paralumbar fossa level. Orchiectomy was performed *via* a ventral approach, by bilateral midline incisions of scrotal skin above the testes. Following surgery, all bats were administered flumazenil for midazolam reversal, and meloxicam for postoperative analgesia, subcutaneously. All bats recovered from anesthesia uneventfully. Bats were monitored for complications up to 10 days following surgery, when skin sutures were removed. No morbidities or mortalities occurred during this period in any of the bats. In conclusion, ventral approach orchiectomy and dorsal approach ovariectomy using the injectable combination alfaxalone-midazolam-morphine in conjunction with local anesthesia and meloxicam are feasible procedures in Egyptian fruit bats and can be performed with relative ease. However, further studies using these techniques in a larger group of bats should be performed to establish their safety.

## 1. Introduction

Egyptian fruit bats, *Rousettus aegyptiacus* (*R. aegyptiacus*), are a common display species in zoological parks, and due to traits like longevity, robust immune system, echolocation and high brain to body ratio, this species is studied in laboratory settings (1). In captivity, these bats breed well throughout the year, and offspring are born every month (2). Conception rates are between 80 and 90% for this species in the wild (3), and gestation has been estimated to be 4 months based on field data (4) and 127 days based on captive observations (Dr. Tal, unpublished data). Generally, one young is born at a time (3, 4), but twins have been reported (2, 5).

Surgical contraception may aid in prevention of overpopulation in the wild and in captivity (6). Additionally, the procedure is essential to study the reproductive system of this species. Another justification for neutering bats is prevention of inbreeding in captivity as a potential factor for hereditary disorders, such as hemochromatosis, a frequent cause of liver disease and mortality in captive *R. aegyptiacus* (7).

### 1.1. Anesthesia

Inhalation anesthesia is used frequently in bats, as it provides quick induction and recovery and is considered to be safe (8). However, due to their small size, leak around the face mask or endotracheal tube can easily occur (9).

Alpha-2-adrenergic agonists were reported in bat species and are usually combined with other drugs, such as ketamine, opioids and/or benzodiazepines (1, 8, 10, 11). A recent study compared dexmedetomidine-butorphanol-midazolam (0.04–0.3–0.3 mg/kg; DBM) and dexmedetomidine-ketamine (0.04–7 mg/kg; DK) in *R. aegyptiacus* undergoing gonadectomy (10).

Alfaxalone-2-hydroxpropyl-β-cyclodextrin (alfaxalone-HPCD) is a synthetic neurosteroid anesthetic agent acting as a γ-aminobutyric acid receptor A (GABA_A_) agonist. Alfaxalone has a high therapeutic index, short half-life, and minimal cardiovascular effects at clinical dose, thus, it can be used for injured or compromised patients (12–15). Moreover, alfaxalone is effective when administered intravenously, intramuscularly or subcutaneously (15–17). Alfaxalone is licensed worldwide for use in dogs and cats and increasing number of studies using alfaxalone in exotic and wild species reported good anesthetic outcome (18–21).

Benzodiazepines are muscle relaxants and anticonvulsants, which produce their effect by potentiation of GABA_A_ receptor, and are considered to have mild cardiopulmonary effects at clinical doses. The benzodiazepine midazolam is water-soluble and is well-absorbed following intravenous, intramuscular or subcutaneous administration (22, 23). In dogs, midazolam was reported to improve induction quality and increase anesthetic duration following intravenous alfaxalone (22, 24). And the combination of alfaxalone-midazolam was recently described in *R. aegyptiacus* (25).

### 1.2. Reproductive system anatomy

The female uterus is duplex, symmetrical, and the two uterine horns are externally caudally united, but their lumina open at the vagina by separate cervical canals. During pregnancy, a single corpus luteum is at the same side of the reproductive tract as the developing embryo, whereas the contralateral ovary contains primary and secondary follicles. The corpus luteum of pregnancy persists until the next ovulation (26–28).

Male subadults testes are abdominal, whereas those of adults are scrotal or mobile between the abdomen and the scrotum through the inguinal canal (29). The male reproductive cycle was reported to be synchronized with that of the females.

### 1.3. Gonadectomies

In fruit bats, a ventral midline approach to ovariohysterectomy was reported (10). However, it may lead to self-mutilation and evisceration. A dorsal approach ovariectomy was described in rodents, and is considered to be very quick and easy to perform (30).

In small mammals, a scrotal approach to castration is described and routinely used (30). In bats, the ductus deferens can be difficult to identify because it is surrounded by fat, therefore, vasectomy was evaluated (6).

The objective of this report is to describe the anesthetic and analgesic management and the surgical procedures of gonadectomy in six adult *R. aegyptiacus* that were presented for ovariectomy or orchiectomy. To the authors' knowledge, none of the reported procedures was described previously in this species.

## 2. Cases description

### 2.1. Animals

Six healthy adult (age unknown) *R. aegyptiacus*, four females and two males, were presented to the Koret School of Veterinary Medicine, Veterinary Teaching Hospital (KSVM-VTH) for ovariectomy and orchiectomy, respectively. Bats were housed at a bat colony at the Zoological Garden of Tel-Aviv University and were brought to KSVM-VTH *via* bat carriers shortly prior to surgery. Acclimatization was not performed, as it was a short procedure and bats were returned to the bat facility following recovery from anesthesia. Due to lack of an appropriate fine weight scale, bats' body weight was estimated to be 130 g (females) and 150 g (males) based on the authors' experience working with this species.

### 2.2. Anesthesia and analgesia

Anesthesia was induced with a combination of 15 mg/kg alfaxalone (Alfaxan, Jurox; Rutherford, Australia; 10 mg/mL), 1.5 mg/kg midazolam (Midolam 5, Rafa Laboratories; Jerusalem, Isreal; 5 mg/mL) and 1.5 mg/kg morphine (Morphine hydrochloride, Aguettant; Lyon, France; 10 mg/mL) combined in an insulin syringe (BD Micro-Fine [0.5-mL, 30-guage, 8 mm], Becton, Dickinson and Company; New Jersey, USA) administered subcutaneously between the shoulder blades while bats were gently restrained covered with a drape. Following injection, bats were monitored every minute for relaxation signs (head relaxation and decreased wing muscle tone), while held under the drape. First effect appeared 4 ± 1 (mean ± SD; range 3–5) min following injection. Mean time to recumbency without movement (i.e., induction time) was 7 ± 1 (5–8) min, although 10 min from injection, one female still showed an occasional ears and wings movement. As anesthetic depth was considered light, the anesthetist decided to administer an extra dose of alfaxalone (5 mg/kg) at 11 min after the initial injection. Mobility then ceased within 2 min. During anesthetic induction, four of the six bats displayed muscle twitching, that subsided after several minutes as anesthetic depth increased.

**Figure 1:**
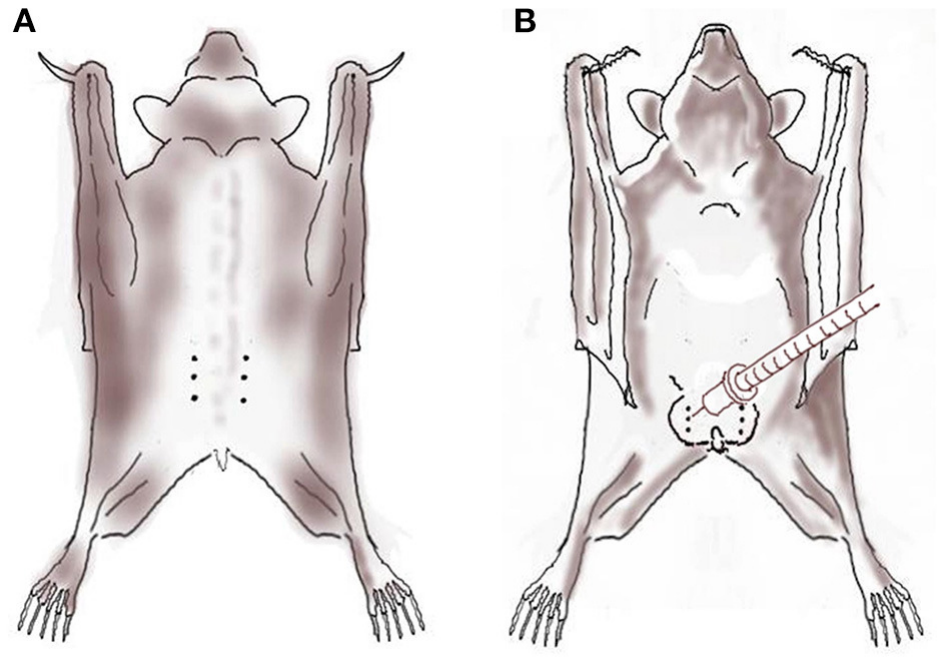
Local anesthesia using local anesthetic solution infiltration (dotted line) over the planned incision line and intratesticular block (syringe) with bupivacaine 0.5% diluted with sodium chloride 0.9% in six Egyptian fruit bats anesthetized with alfaxalone-midazolam-morphine prior to ovariectomy [**(A)**; female; dorsal view] and orchiectomy [**(B)**; male; ventral view].

Following aseptic preparation, bupivacaine 0.5% solution (Kamacaine 0.5%; Kamada, Beit-Kama, Israel) diluted in sodium chloride 0.9% (B Braun Melsungen AG, Melsungen, Germany; 1:1 volume ratio; final concentration 0.25%) was infiltrated using an insulin syringe (BD Micro-Fine, 0.5-mL, 30-guage) at the planned incision line prior to surgery (Figure 1). In the males, diluted bupivacaine was also injected intratesticularly as described in cats and dogs (31, 32). The total volume of diluted bupivacaine injected was 0.15 mL in the females (2.5–3 mg/kg; divided equally between left and right incision lines), and 0.2 mL in the males (3.5 mg/kg; divided equally to four: bilateral scrotal incisions and bilateral testicles).

During the procedure, anesthetic depth and physiologic variables were monitored every 5 min. Isoflurane was available for rescue anesthesia in case anesthetic depth was considered inadequate. Heart rate (HR) and rhythm were monitored using a doppler ultrasonic flow detector (Parks Medical Electronics, Inc.; Oregon, USA) placed and taped on the sternum at the heart level. Respiratory frequency (*f*_R_) was measured by observation of thoracic movements. Body temperature was measured using rectal thermometer. Anesthetic depth was considered appropriate in all bats (flaccid wing muscle tone, absent or reduced palpebral reflex and jaw tone), and no movement or elevation in heart or respiratory rates in response to surgical stimulation occurred. Physiologic variables and anesthetic complications are presented in Table 1. Sudden decrease in heart rate was noted in response to pulling on the gonads during surgery in two bats (one female and one male), which was short-lived and resolved without treatment.

**Table 1:** Ranges (minimum-maximum) of physiologic variables recorded from six Egyptian fruit bats anesthetized with alfaxalone-midazolam-morphine for gonadectomy.

	**Heart rate (beats per minute)**	**Respiratory frequency (breaths per minute)**	**Rectal temperature (degrees Celsius)**
Female 1	260–320	16–30	34.5
Female 2	360–400	12–20	34.9
Female 3	164–300^*^	30–30	34.2
Female 4	220–400^**^	8–28^**#**^	33.9
Male 1	180–360^*^	10–20	34.2
Male 2	180–200	20–40^**#**^	35.8
Median	**200–340**	**14–29**	**34.4**

Rectal temperature is reported immediately at the end of surgery.

^*^Sudden decrease in heart rate (female 2, from 280 to 200; male 1, from 300 to 180) in response to pulling on the gonads.

^**^Arrhythmia of several dropped beats was heard for a period of ~2 min during preparation for surgery. Electrocardiogram monitor for animals < 500 gram body weight was not available.

^**#**^Irregular breathing pattern observed.

The last row (in bold) provides the medians of minimum and maximum values from all bats.

Bats were placed on an insulated operating table and oxygen was supplemented (1 L/min) *via* a small non-fitted facemask throughout the procedure. Three mLs of warm lactated Ringer's solution (Teva Medical, Petah Tikva, Israel) were administered subcutaneously, and eye lubrication was applied (DuraTears, Alcon-Couvreur; Puurs, Belgium). During surgery, bats were actively warmed with a forced-air warming system (Thermacare; Gaymar Industries Inc.; New York, USA).

### 2.3. Ovariectomy (dorsal approach)

A dorsal approach was used to prevent post-operative self-mutilation. Bats were placed in ventral recumbency, and the dorsal skin was shaved and aseptically prepared over the thoraco-paralumbar region and draped. A skin incision (1–1.5 cm) was made ~1 cm lateral to the midline, at the paralumbar fossa region, in a cranial to caudal direction (Figure 1). The fat pad underlining the body wall was retracted and the musculature underneath it separated to enable visualization of the reproductive tract. The ovarian blood vessels were ligated using absorbable ligatures (3-0 Chromic catgut, Ethicon, New Jersey, USA). The ovary, oviduct and 2 cm of the cranial uterine horn were clamped with hemostatic forceps and excised using Metzenbaum scissors. Care was taken not to touch the kidneys, which lie adjacent to the ovaries and tend to bleed profusely when injured.

In two females, moderate non-specific local hemorrhage of the tissues around the ovary occurred. Local pressure was applied using sterile wooden cotton swabs until the bleeding subsided. The muscle layer was sutured using absorbable ligatures (3-0 PDS II, Ethicon) with a simple continuous pattern, following replacement of the fat pad. Simple continuous subcutaneous sutures (3-0 PDS II, Ethicon), and intradermal continuous sutures (3-0 PDS, Ethicon) were placed. Finally, for extra protection against mutilation, three to four nylon sutures were placed in the skin in a simple interrupted pattern (3-0 Nylon Assut Medical Sàrl, Pully-Lausanne, Switzerland). Mean ovariectomy time (from first incision to completion of the final suture) was 31 ± 5 (26−37) min.

### 2.4. Orchiectomy (ventral approach)

Scrotal skin was aseptically prepared and draped. The testis was manually held slightly distal to the body in order to prevent its retraction into the abdomen. A 5–8-mm longitudinal ventral scrotal incision was made through the skin, subcutaneous tissue and vaginal tunics, using number 15 scalpel blade. The testis was exteriorized and the epididymis, ductus deferens, testicular artery and vein (pampiniform plexus) identified. The ductus deferens and testicular vessels were clamped using mosquito hemostats, and three circumferential ligatures (4-0 PDS, Ethicon) were placed 0.5 cm apart around them. The ductus deferens and testicular vessels were dissected distal to the distal ligature, to disconnect the testis and epididymis from the body. Scrotal skin was sutured using nylon ligatures (3-0 Nylon Assut Medical Sàrl). Orchiectomy time (from first incision to completion of the final suture) was 7–10 min, and bleeding was minimal.

### 2.5. Recovery, postoperative analgesia, and follow-up

Two female bats began flickering their ears during placement of the last skin suture (at 49 and 50 min following anesthetics injection). In order to facilitate recovery, at the end of surgery bats were administered 0.3 mg/kg flumazenil (Flumazenil 0.1 mg/mL, Mylan S.A.S.; Saint Priest, France) subcutaneously to antagonize the midazolam (25).

Total anesthesia time (from anesthetics injection to antagonist administration) was 59 ± 7 (52–68) min for ovariectomies and 28–37 min for orchiectomies. Recovery time (from flumazenil administration until climbing to the carrier roof) was 30 ± 5 (22−35) min. Recovery time in the present report was longer than reported for dexmedetomidine-based protocols (DK 5.8 ± 2.5; DBM 7.1 ± 3.5 min) (10). This difference could be attributed to differences in drug protocols, surgery duration and definitions of recovery time. Once recovered from anesthesia bats were offered commercial mango juice orally. They were all warmed with a heating lamp, and body temperature was monitored until it reached 36°C.

For postoperative analgesia, bats were administered a subcutaneous injection of 1 mg/kg meloxicam (Metacam, Boehringer Ingelheim Vetmedica Inc., St. Joseph, MO, USA) at the end of surgery, and then continued with 1 mg/kg oral meloxicam (Loxicom oral suspension, Norbrook Laboratories Limited, Newry, Northern Ireland) every 24 h for two more days.

Following complete recovery from anesthesia, bats were taken back to the bat facility and housed in dark, temperature and humidity-controlled conditions. Fruits and water were provided in accordance with recommendations for the species. Bats were individually housed in order to prevent picking and tearing the sutures by other bats. Bats underwent veterinary exam daily and skin sutures were removed after 10 days. No morbidities or mortalities occurred during this period in any of the bats.

## 3. Discussion

The present report describes the feasibility of performing gonadectomy in *R. aegyptiacus* using a surgical approach and anesthesia and analgesia regime, including local anesthesia, not previously reported in this species.

### 3.1. Anesthesia and analgesia

Injectable anesthesia was preferred over inhalant anesthesia in the present report, as scavenging and control of inhalant excess in very small animals is poor (i.e., tracheal tube cannot be sealed), which result in personnel exposure (1, 9). Several injectable anesthetics were described in bats for various procedures; however, specific controlled studies are relatively sparse. In bats, medetomidine- or dexmedetomidine-based protocols were reported to provide deep sedation or anesthesia (1, 8, 10, 11). Alpha-2-adrenergic agonists tend to produce significant effects on the cardiovascular system (initial hypertension, profound reflex bradycardia and poor peripheral circulation) (11, 33, 34), therefore, they were avoided in the present report. A recent study compared DBM and DK in *R. aegyptiacus* undergoing gonadectomy. Both protocols provided quicker induction compared with the present report (DK 2.5 ± 2.8; DBM 2.8 ± 0.8 min), although, both protocols required isoflurane supplementation during surgery (mean inspired fraction of 0.7%) (10).

The successful experience with alfaxalone-midazolam anesthetic combination in *R. aegyptiacus* by the authors (25) have led us to use this combination in the present report. However, neither alfaxalone nor midazolam have analgesic properties (15, 23), therefore, morphine, a pure μ-opioid receptor agonist, was added to the anesthetic combination in order to provide preemptive analgesia (34). Controlled studies reporting the analgesic effects of opioids in bats are lacking. Thus, the use of morphine during bat gonadectomies was based on the reported use and dose of morphine in *R. aegyptiacus* (1), and on the description that it provides analgesia in bats (8).

Multimodal analgesia refers to administration of several analgesic drug classes or techniques with different mechanisms of action to target different receptors along the nociceptive pathway. This approach is considered the best practice in pain management both in human and veterinary medicine, due to the synergistic effects that optimize analgesia (14, 35). In the present report, morphine, meloxicam, and local anesthesia were used to provide multimodal analgesia. Inclusion of local analgesic technique is a key component of multimodal pain management, as local anesthetics inhibit nerve impulse conduction to the spinal cord and higher nervous system centers *via* sodium-channel blockade (14, 35). Although not reported in bats, the authors applied local anesthetic techniques previously reported in other species during gonadectomies (31, 32), to provide better analgesia and to increase animal welfare. Although locoregional techniques were not used by Amari et al. it was recommended by the authors for bat gonadectomies (10).

Telemetrically measured resting HR and *f*_R_ in *R. aegyptiacus* were reported to be 226 ± 28 beats per minute and ~40 breaths per minute, respectively, when in the thermoneutral zone (36). In some of the bats in the present report, HR and *f*_R_ were lower than the reference range, which may indicate some cardiopulmonary depression, as reported in bats under general anesthesia (1, 11). Compared with alfaxalone-midazolam anesthesia (25), some HR and *f*_R_ measurements were lower in the present report. These lower values may have been attributed to morphine addition, as opioids tend to increase vagal tone and decrease HR (34). Alfaxalone-midazolam-morphine protocol in the present report produced higher HR than dexmedetomidine-based protocols (DK 181 ± 31 bpm; DBM 203 ± 47 bpm). However, *f*_R_ was lower (DK 112 ± 26 rpm; DBM 85 ± 21 rpm) (10). Although bats were actively warmed during surgery, the immediate postoperative rectal temperature was below 36.5 °C, which is the average normal rectal temperature reported for *R. aegyptiacus* (36). This emphasizes the significant heat loss of small mammals such as bats under anesthesia and the importance of providing insulation and active warming during the procedure.

Twitching during induction of anesthesia was observed in several bats and was previously reported in *R. aegyptiacus* under alfaxalone-midazolam (25) or medetomidine-midazolam based combinations (1). This adverse effect was short-lived and disappeared with increased anesthetic depth. It was previously hypothesized that this observation may be characteristics of *R. aegyptiacus* injectable anesthesia, due to firstly inhibition of inhibitory neurons, resulting in muscular contractions and later additional inhibition of excitatory neurons, resulting in muscular relaxation (25, 37).

Abnormal heart rhythm and irregular breathing pattern were previously reported in *R. aegyptiacus* under medetomidine-midazolam based anesthetic combinations (1), but not following alfaxalone-midazolam (25). These may have been related to the gonadectomy procedure. The authors are familiar with a sudden tachycardia in response to pulling on the gonads in dogs and cats, due to sympathetic stimulation (31, 32). However, in the present report, decreased heart rate occurred, which may indicate a vagal response in this species.

For recovery, flumazenil was used to reverse midazolam sedation. Flumazenil is a specific benzodiazepine competitive antagonist, which inhibits the central effects of benzodiazepine agonists. Flumazenil is well-tolerated across a wide range of doses and has little side effects (38, 39). Furthermore, flumazenil administration following alfaxalone-midazolam anesthesia significantly shortened recovery time and increased recovery quality in *R. aegyptiacus* in comparison to saline control (25).

### 3.2. Ovariectomy

Ovariectomy using a ventral, lateral or dorsal approach was described in numerous species including equine, canine, feline and rodents (40–42). Ovariectomy performed on three vampire bats (*Desmodus rotundus*), was carried out using a ventral midline incision and all bats recovered uneventfully. Surgical time was longer than the time in the current report and lasted 45–56 min (43). Another recent study performing ventral approach gonadectomies in *R. aegyptiacus* reported longer mean surgical times; 44–53 min, but specific surgical duration of ovariectomy vs. orchiectomy was not reported (10). The main advantage of the ventral midline approach is the possibility to visualize the entire reproductive tract during surgery. However, in obese bats or ones with a post prandial satiated gastrointestinal tract, it can be challenging to visualize parts of the reproductive tract and perform an ovariectomy *via* the ventral approach. Using the dorsal approach, most of the reproductive tract can be viewed: ovaries, oviducts, uterine horns, and pregnancy, however, the uterine body is difficult to visualize. The advantage of the dorsal approach is that the ovaries are located immediately below the incision site and are easily removed. Additionally, in the authors' experience, *R. aegyptiacus* females are capable of eviscerating and mutilating internal organs after ventral laparotomy (Dr. Tal, unpublished data). It is possible that due to the pecking nature of fruit bats they are prone to postoperative self-mutilation compared with vampire bats. The potential surgical complications posed when using the ventral ovariectomy approach, make the dorsal approach potentially superior in *R. aegyptiacus*, as in the present report surgical time was shorter, and complications seemed to be minimal. However, this technique should be tested in a larger group of bats to validate its safety.

### 3.3. Orchiectomy

Ventral approach orchiectomy technique as described in the present report, can be easily used in this species. Testes were retrieved with ease from the abdominal and pre-scrotal positions, vessels were ligated in a simple manner and no potential complications during or following surgery were observed.

Two other techniques of orchiectomy were described in a study in other species of fruit bats. In that study, open orchiectomy by transfixing ligation of the spermatic cord was performed in 14 Ruwenzori long-haired fruit bats (*Rousettus lanosus*), and orchiectomy with radiosurgery was performed in 125 Jamaican fruit-eating bats (*Artibeus jamaicensis*), and one Ruwenzori bat, under general anesthesia. Total surgery time for open orchiectomy was much longer than the total surgery time in the present report (26–38 min compared with 7–10 min). Postoperative complication (prolonged recovery) occurred in 1/14 bats. Radiosurgeries were short, lasting 5–7 min, however, major complications occurred; 4.8% morbidity rate (dyspnea, hemorrhage, and prolonged recovery), and 1.6% mortality rate (2 bats) (44). Complications may have been common following radiosurgery due to the large cohort size, although the technique itself may also play a role.

Another study described vasectomy performed in nine *R. aegyptiacus* males. The authors reported that it was difficult to identify the mesoductus deferens, and postoperatively 27% of the testes appeared to adhere to the scrotal sac for at least 14 months. Otherwise, the technique appeared quick (6). Although vasectomy renders the bats sterile, it does not eliminate full steroid hormonal activity and testosterone, as opposed to orchiectomy. Depending on the population control goals, the appropriate surgical technique should be used.

### 3.4. Limitations

Limitations to this case report include the small number of animals, and lacking the ability to monitor blood pressure, hemoglobin oxygen saturation, electrocardiogram, end tidal CO_2_, and continuous rectal temperature. Postoperative pain evaluation was not performed, and to the authors' knowledge, there are no specific pain scales for bat species. For this reason, meloxicam was administered postoperatively regardless of pain evaluation. Additionally, the inability to accurately weigh the bats is a major limitation of this report. In very little animals where weight estimation is challenging, small errors can lead to large differences in drug doses.

## 4. Conclusion

In summary, ventral approach orchiectomy and dorsal approach ovariectomy using the injectable combination alfaxalone-midazolam-morphine in conjunction with local anesthesia and meloxicam are feasible procedures in *R. aegyptiacus* bats and can be performed with relative ease. However, further studies comparing this anesthesia regimen with other protocols and assessing the reported surgical procedures in a larger group of bats should be performed to establish their safety.

## Data availability statement

The raw data supporting the conclusions of this article will be made available by the authors, without undue reservation.

## Ethics statement

This case series was carried out as a standard treatment in accordance with good clinical practice. Written informed consent was obtained for including the animal in this case series.

## Author contributions

ST and YS-B contributed to the data acquisition and interpretation, drafted and revised the manuscript, and approved the final version to be submitted.
